# Delayed access to feed early post-hatch affects the development and maturation of gastrointestinal tract microbiota in broiler chickens

**DOI:** 10.1186/s12866-022-02619-6

**Published:** 2022-08-24

**Authors:** Monika Proszkowiec-Weglarz, Katarzyna B. Miska, Laura E. Ellestad, Lori L. Schreier, Stanislaw Kahl, Nadia Darwish, Philip Campos, Jonathan Shao

**Affiliations:** 1 United States Department of Agriculture (USDA), Agricultural Research Service (ARS), Northeast Area (NEA), Animal Biosciences and Biotechnology Laboratory (ABBL), Beltsville, 10300 Baltimore Avenue, B-200, Rm. 100B, BARC-East, Beltsville, MD 20705 USA; 2 Department of Poultry Science, University of Georgia, Athens, GA 30602 USA; 3 United States Department of Agriculture (USDA), Agricultural Research Service (ARS), Northeast Area (NEA), Statistic Group, Beltsville, MD 20705 USA

**Keywords:** Chicken, Ileum, Ceca, Microbiota, 16S, Delayed access to feed

## Abstract

**Background:**

The first two weeks of post-hatch (PH) growth in broilers (meat-type birds) are critical for gut development and microbiota colonization. In the current broiler production system, chicks may not receive feed and water for 24 to 72 h due to variations in hatching time and hatchery management. Post-hatch feed delay affects body weight, feed efficiency, mortality, and gut development. The goal of this study was to investigate changes in the microbiome in broiler chickens early PH and the effect of delayed access to feed on the microbiota.

**Results:**

Chicks either received feed and water immediately after hatch or access to feed was delayed for 48 h to mimic commercial hatchery settings (treatment, TRT). Both groups were sampled (*n* = 6) at -48, 0, 4 h, and 1 (24 h), 2 (48 h), 3 (72 h), 4 (96 h), 6 (144 h), 8 (192 h), 10 (240 h), 12 (288 h) and 14 (336 h) days PH. Ileal (IL) and cecal (CE) epithelial scrapings (mucosal bacteria, M) and digesta (luminal bacteria, L) were collected for microbiota analysis. Microbiota was determined by sequencing the V3-V4 region of bacterial 16S rRNA and analyzed using QIIME2. The microbiota of early ileal and cecal samples were characterized by high abundance of unclassified bacteria. Among four bacterial populations (IL-L, IL-M, CE-L, CE-M), IL-M was the least affected by delayed access to feed early PH. Both alpha and beta diversities were affected by delayed access to feed PH in IL-L, CE-M and CE-L. However, the development effect was more pronounced. In all four bacterial populations, significant changes due to developmental effect (time relative to hatch) was observed in taxonomic composition, with transient changes of bacterial taxa during the first two weeks PH. Delayed access to feed has limited influence on bacterial composition with only a few genera and species affected in all four bacterial populations. Predicted function based on 16S rRNA was also affected by delayed access to feed PH with most changes in metabolic pathway richness observed in IL-L, CE-L and CE-M.

**Conclusions:**

These results show transient changes in chicken microbiota biodiversity during the first two weeks PH and indicate that delayed access to feed affects microbiota development. Proper microbiota development could be an important factor in disease prevention and antibiotic use in broiler chickens. Moreover, significant differences in response to delayed access to feed PH between luminal and mucosal bacterial populations strongly suggests the need for separate analysis of these two populations.

**Supplementary Information:**

The online version contains supplementary material available at 10.1186/s12866-022-02619-6.

## Background

The chicken gut microbiota, composed of bacteria, fungi, viruses and protists, is characterized by commensal, symbiotic, and pathogenic relationships with its host [1]. Bacterial species identified in the broiler (meat-type chicken) gastrointestinal tract (GIT) [2] play an important role in host nutrition, including feed digestion, nutrient absorption and metabolism, pathogen exclusion, endocrine activity, immune system development and functioning, and performance efficiency [3, 4]. In broiler chickens, the symbiotic relationship between the host and the microbiota has been characterized by nutrient exchange, modulation of the immune system, GIT physiology and pathogen exclusion [2, 5–8].

For a long time, the GIT of birds has been considered sterile at hatch [9, 10]. Recent studies indicate that the GIT contains a microbial population that could be acquired through translocation through pores in the eggshell prior to cuticle deposition [11, 12]. In addition to horizontal transmission by penetration through the eggshell [13], some microbiota of the maternal oviduct could be vertically transmitted to the embryo. The bacteria might be directly deposited into the yolk, albumin, eggshell membrane and eggshell during egg formation before oviposition as a result of the presence of microbiota in the oviduct [11, 14]. Even though strict hygiene practices reducing microbial load are implemented, the incubator environment is the first bacteria source for newly hatched chicks [15]. Immediately after hatch, birds passively or actively acquire microbiota from the environment. In comparison to mammals, there is a limited influence of parental microbiota [16]. Instead, the first microbiota that the chicks are exposed to comes from non-avian sources such as the incubator, animal handlers, bedding material, and transport boxes [17]. This random microbiota colonization of newly hatched chicks by environmental organisms leads to high sensitivity to infections such as pathogenic *E. coli*, *Clostridium perfringens,* or *Salmonella* [18, 19]. In farms, chickens are exposed to highly diverse microbiota in litter, feed, water, and air. The development of microbiota that occurs as a process of ecological succession can be influenced by age, host genotype and sex, diet composition and form, dietary additives such as probiotics, prebiotics, symbiotics, phytobiotics and bacteriophages, stress, antibiotics, and location in GIT [4, 5, 7, 20–22]. Due to variable oxygen and nutrient availability, and changes in pH along the GIT, significant differences in bacterial communities between small and large intestine as well as between parts of the small intestine have been detected [23]. Moreover, differences between communities associated with mucosal surface and within the GIT lumen have been shown [24, 25].

In comparison to other animals, the avian microbiota is characterized by a relatively low diversity due to the rapid transition of food through the digestive tract and short retention time [5]. Intestinal microbiota reaches a relatively mature community state between weeks 2 and 3 of PH development [26]. The mature chicken microbiota is characterized by 10^11^ CFU/g in the hindgut, 10^8^ CFU/g in the ileum and 10^3^–10^4^ CFU/g in the stomach, duodenum and jejunum [6, 23]. Between 200–350 different bacterial species have been detected in the enteric microbiota of individual chickens, while over 640 different bacterial species have been detected so far in the chicken GIT [27, 28].

In the current commercial broiler management chicks can remain without access to feed or water for up to 72 h PH [29–33] due to a 24–36 h hatch window and removal of the chicks from the hatchery occurring at the same time [29, 34], PH selection, vaccination, sexing, sorting and transportation to the farms. Prolonged lack of access to feed and water early PH has detrimental effects on body weight at placement [29], organ weight [35–37], PH growth in early hatched chicks [38–40], feed conversion ratio, mortality, and GIT development [32]. We have shown previously that 48 h delay in access to feed PH results in inhibition of the up-regulation of lipogenic genes and lipogenic transcription factor genes [41], as well as is affecting the expression pattern of the following group of genes: Ca and P transporter genes [42], small intestine gut barrier and tight-junction-related genes [43], ceca development-related genes [44] and carbohydrate and amino acids utilization genes [45]. Within the muscle, hormonal signaling, cellular differentiation and protein metabolism genes were also affected due to delayed access to feed [46].

The purpose of this study was to perform a comprehensive analysis of the ontogeny of broiler chicken intestinal microbiota and to determine the effect of delayed access to feed for the first 48 h PH on microbiota in luminal (L) and mucosal (M) bacterial population of ileum (IL) and ceca (CE) during the first two-weeks PH. A better understanding of developing microbiota in the GIT has been suggested to have high potential in improving microbiome management practices, and the health and well-being of broiler chickens [47]. Moreover, Jurburg et al. [48] have confirmed that the first week after hatch is the most critical to broiler microbiota development and that early colonization of the GIT is very important for poultry health and productivity [49].

## Results

### Sequencing characteristics

A total of 53,683,603 sequence reads were obtained from 384 ileal and cecal samples. The total and mean number of raw sequencing reads as well as the number of reads per specimen after quality trimming (IL, CE, IL-L, IL-M, CE-L and CE-M) are presented in Table 1. Due to significant differences in the number of sequencing reads between samples collected from -48 to 4 h PH and samples collected from 1 day PH onward, the first group of samples (-48–4 h PH, IL and CE) was analyzed separately. Rarefaction curves indicated that high coverage was achieved in all samples analyzed (Fig. S1a-e) and they were used to determine rarefaction depth for subsequent diversity analysis (Table 1).

**Table 1:** Sequencing information for ileal (IL) and cecal (CE) luminal (L) and mucosal (M) samples^a^

	Sequencing data				
	IL & CE^b^	IL-L^c^	IL-M^c^	CE-L^c^	CE-M^c^
Raw reads					
Total	203,099	18,944,578	4,556,341	11,065,680	18,913,905
Mean	4,615	217,753	54,895	125,746	230,657
Reads after quality trimming					
Total	12,568	6,841,576	1,764,019	2,612,896	7,437,805
Mean	285	78,639	21,253	29,692	90,704
Sequencing depth for analysis	119	1267	1357	2,899	11,510

^a^*n* = 6 for each time point and each section

^b^Samples collected from -48 h to 4 h post-hatch from ileum and ceca

^c^Samples collected from days 1 (24 h) to 14 (336 h) post-hatch

### Early microbiota

**Fig. 1:**
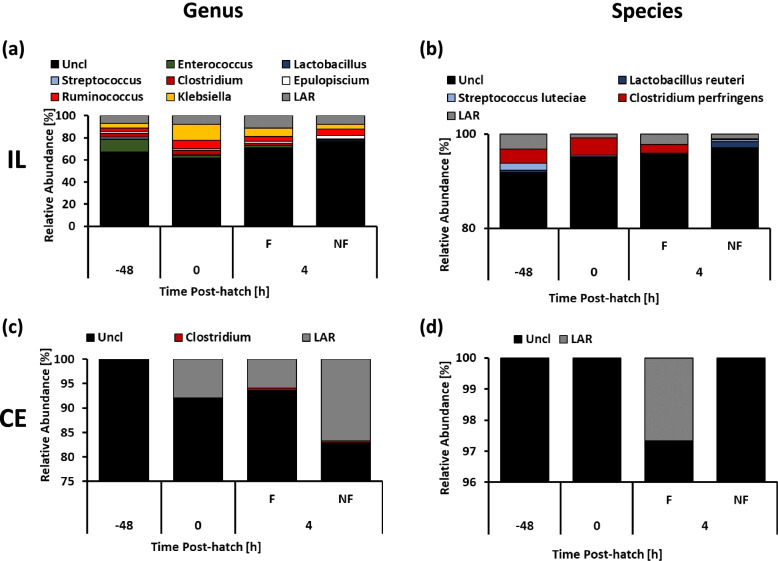
Taxonomic profile [relative abundance (%)] in ileal (IL, **a**-**b**) and cecal (CE, **c**-**d**) bacterial populations at the genus and species level in embryo (-48 h), chicks at hatch (0 h) and 4 h post-hatch. Uncl – unclassified bacterial reads, Low Abundant Reads (LAR). 4 h post-hatch chicks have access to feed (F) or do not have access to feed (NF)

Due to the relatively low number of reads from samples collected from -48 h to 4 h PH, these samples were analyzed separately from the data set collected from 1 day PH onward. The number of Amplicon Sequence Variants (ASVs), richness, evenness and Shannon index were not affected (*P* > 0.05) by time PH or experimental conditions PH, while significant (*P* < 0.005) differences for all four indices were detected for tissues comparison (ileum vs. cecum, data not shown). The number of ASVs, richness, evenness and Shannon index were higher in ileal samples in comparison to cecal samples collected from birds between -48 h and 4 h PH (data not shown). Similarly, beta diversity was only affected (*P* < 0.001) by the source of samples (ileum vs. cecum). Taxonomic composition of the samples collected early is presented in Fig. 1. Over 60% and 90% of the ileal microbiota in the embryos (-48 h PH) and early PH birds (up to 4 h PH) were identified as unclassified bacteria at the genus and species level, respectively (Fig. 1a and b). The ileum of the embryo and early PH chicks was also colonized by genera *Enterococcus*, *Clostridium*, *Ruminococcus*, *Klebsiella* and LAR (bacterial low abundance reads) while *Clostridium perfringens*, *Streptococcus luteciae,* and *Lactobacillus reuteri* were the only species identified in ileum samples at this stage of development (Fig. 1a and b). In contrast to IL, CE microbiota at early stages of development was characterized mostly by a high abundance of unclassified bacteria and LAR at both, the genus and species level (Fig. 1c and d). A relatively low level of *Clostridium* was present in chicks at 4 h PH (Fig. 1c).

### Alpha diversity

**Fig. 2:**
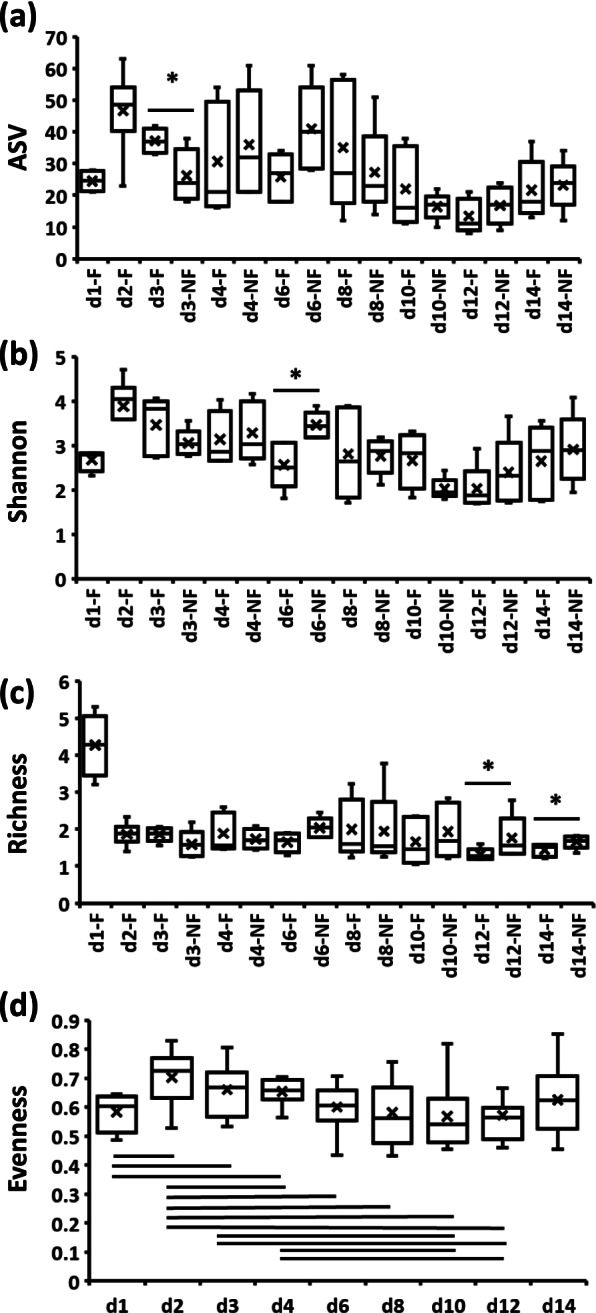
Effect of delay in feed access for the first 48 h post-hatch on alpha diversity indices (**a**) number of amplicon sequence variants (ASV), (**b**) Shannon index, (**c**) Richness and (**d**) Evenness in ileal luminal bacterial population from day 1 (24 h) through day 14 (336 h) post-hatch. When the interaction between time and treatment was significant, only significant (*P* < 0.05) differences between fed (F) and not fed (NF) groups for a single age are shown as indicated by asterisk. When the interaction between time and treatment was not significant, only significant (*P* < 0.05) main effects (time and treatment) are presented and significant differences between groups are indicated by lines

**Fig. 3:**
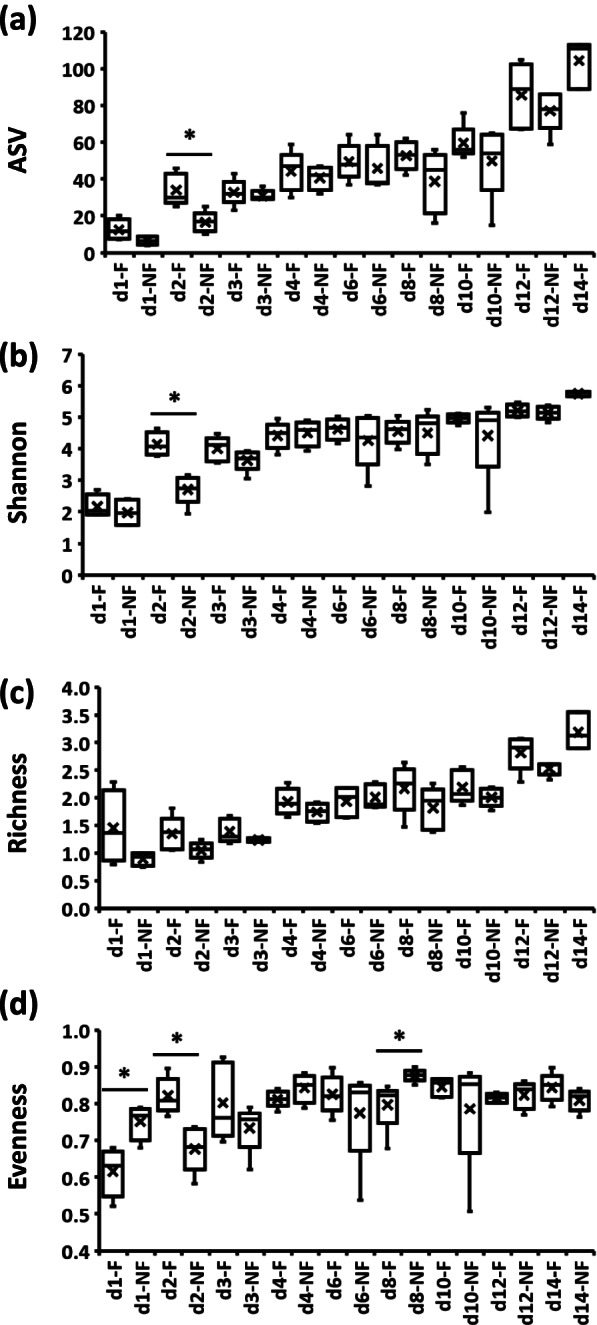
Effect of delay in feed access for the first 48 h post-hatch on alpha diversity indices (**a**) number of amplicon sequence variants (ASV), (**b**) Shannon index, (**c**) Richness and (**d**) Evenness in cecal luminal bacterial population from day 1 (24 h) through day 14 post-hatch. When the interaction between time and treatment was significant, only significant (*P* < 0.05) differences between fed (F) and not fed (NF) group are shown as indicated by asterisk

**Fig. 4:**
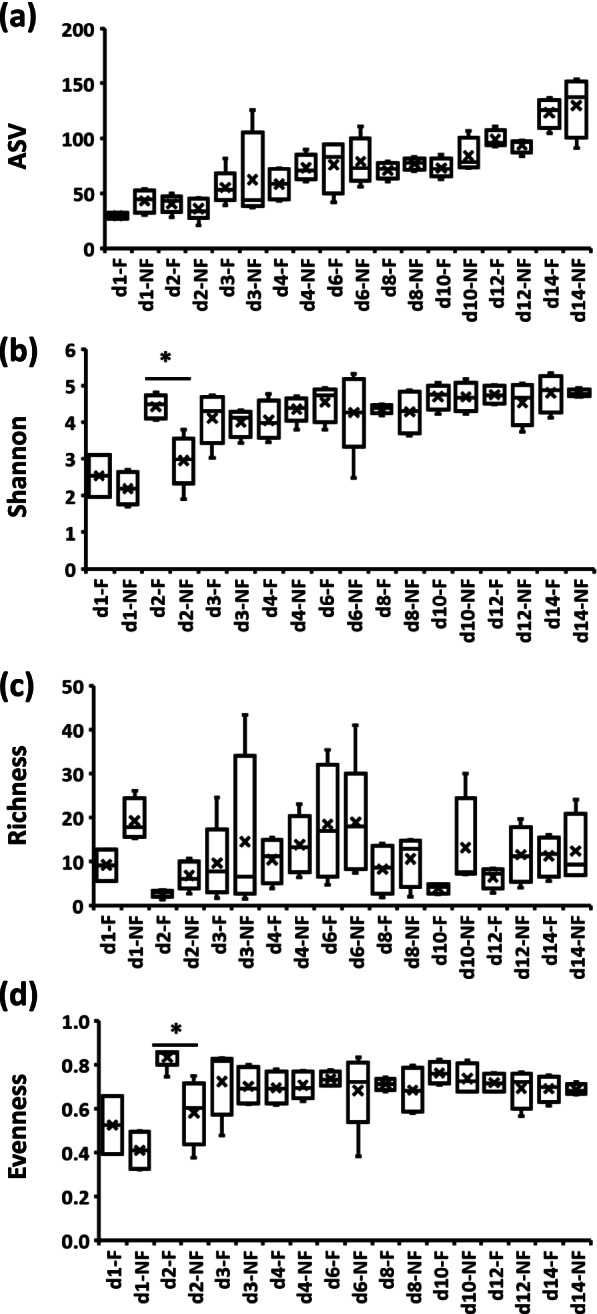
Effect of delay in feed access for the first 48 h post-hatch on alpha diversity indices (**a**) number of amplicon sequence variants (ASV), (**b**) Shannon index, (**c**) Richness and (**d**) Evenness in cecal mucosal bacterial population from day 1 (24 h) through day 14 (336 h) post-hatch. When the interaction between time and treatment was significant, only significant (*P* < 0.05) differences between fed (F) and not fed (NF) group are shown as indicated by asterisk

The effects of time and delayed access to feed PH (TRT) on alpha diversity indices in ileal and cecal bacterial communities from day 1 PH onward are presented in Table 2 and (Figs. 2, 3 and 4). In the bacterial population of IL-L, the number of ASV were significantly (*P* < 0.05) higher in F (feed immediately after hatch) birds in comparison to NF (without access to feed for the first 48 h) birds on day 3 PH (Fig. 2a), the Shannon index was increased in NF birds in comparison to F birds at 6 days PH (Fig. 2b), while the richness of bacterial communities was affected by the delay in access to feed at 12 and 14 days PH (Fig. 2c). On both days, NF birds were characterized by higher richness in comparison to F birds (Fig. 2c). Evenness was only affected significantly (*P* < 0.05) by time, with an increase between day 2 and 4 PH in comparison to day 1, followed by a decrease until day 12 PH in comparison to day 2–4 PH (Fig. 2d). In contrast to IL-L, in IL-M, none of the alpha diversity indices were affected throughout the experiment (Table 2). In CE-L bacterial communities, all four alpha diversity indices exhibited a Time x TRT interaction (Table 2, Fig. 3). Both number of ASVs and Shannon index were significantly (*P* < 0.05) lower in NF birds 2 days PH in comparison to the F birds (Fig. 3a and b, respectively). Even though Time × TRT interaction was significant in CE-L for richness, a pairwise comparison showed no significant differences between F and NF birds for each time point (Fig. 3c). In the case of evenness, NF birds were characterized by higher evenness at 1 and 8 days PH in comparison to F birds, while on day 2 PH, F birds had higher evenness in comparison to NF birds (Fig. 3d). No pairwise comparison was significant between F and NF groups for number of ASV and richness in CE-M microbial population (Fig. 4a and c, respectively) despite a significant Time x TRT interaction (Table 2). Both Shannon index and evenness were significantly higher in F birds in comparison to NF group 2 days PH (Fig. 4b and d, respectively).

**Table 2:** Kruskal–Wallis *p*-values for interactive Time × TRT (fed or delayed access to feed) or main (time and TRT) effects for alpha diversity indices in ileal (IL) and cecal (CE) luminal (L) and mucosal (M) samples collected from days 1 (24 h) to 14 (336 h) post-hatch

Pr = F				
	IL-L	IL-M	CE-L	CE-M
ASV				
Time	< 0.001	0.544	< 0.001	< 0.001
TRT	0.676	0.233	0.196	0.531
Time × TRT	0.002	0.544	< 0.001	< 0.001
Shannon diversity index				
Time	< 0.001	0.884	< 0.001	< 0.001
Trt	0.843	0.707	0.296	0.178
Time × TRT	0.004	0.947	< 0.001	0.003
Faith’s Phylogenetic Diversity (Richness) index				
Time	< 0.001	0.153	< 0.001	0.008
Trt	0.506	0.089	0.177	0.004
Time × TRT	0.002	0.228	< 0.001	0.013
Pielou’s Evenness index				
Time	0.013	0.486	< 0.001	0.032
Trt	0.565	0.964	0.491	0.021
Time × TRT	0.057	0.638	< 0.001	0.026

*ASV* Amplicon sequence variant

### Beta diversity

**Fig. 5:**
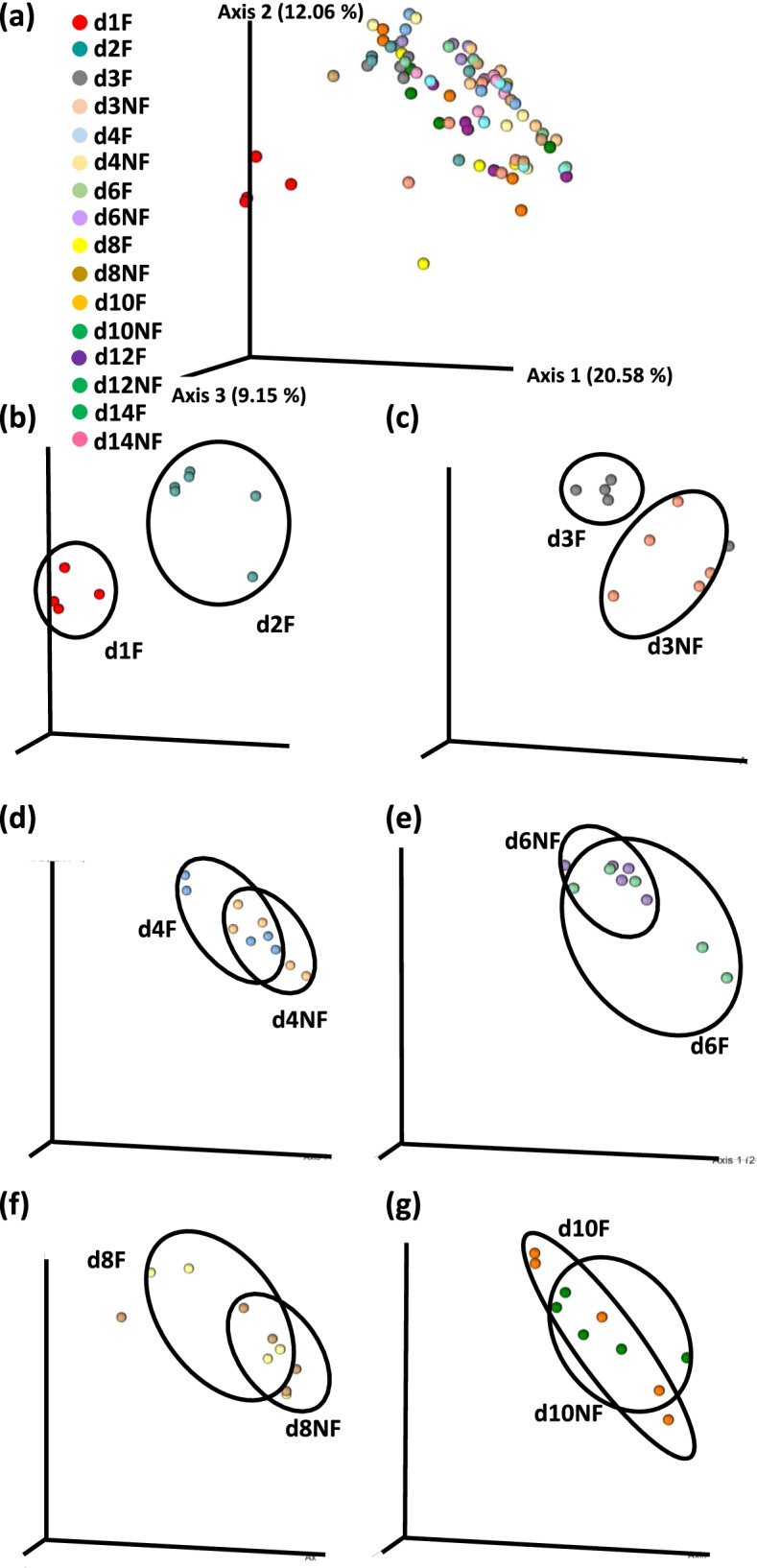
Effect of delay in feed access for the first 48 h post-hatch on beta diversity of ileal luminal bacterial population from day 1 (24 h) through day 14 (336 h) post-hatch using the Principal Coordinate analysis (PCoA) based on the Unweighted UniFrac distances between specific groups. Panel (**a**) shows PCoA for all samples while (**b**-**g**) panels depict differences between treatment groups at (**b**) day (**d**) 1 and 2, (**c**) d 3; (**d**) d 4, (**e**) d 6, (**f**) d 8 and (**g**) d 10 post-hatch. F – chicks fed immediately post-hatch; NF – chicks without access to feed for the first 48 h post-hatch

**Fig. 6:**
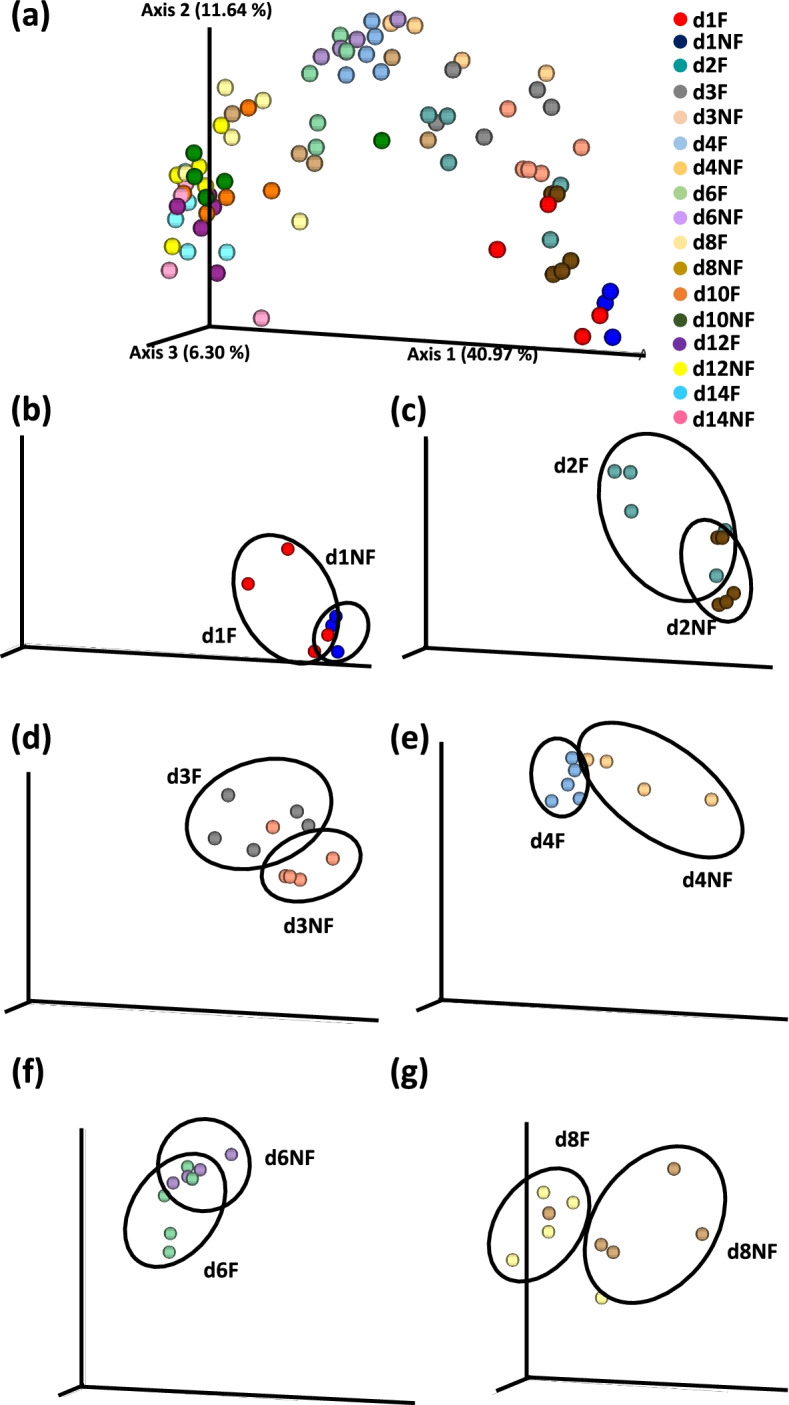
Effect of delay in feed access for the first 48 h post-hatch on beta diversity of cecal luminal bacterial population from day 1 (24 h) through day 14 (336 h) post-hatch using the Principal Coordinate analysis (PCoA) based on the Unweighted UniFrac distances between groups. Panel (**a**) shown PCoA for all samples while (**b**-**g**) panels depict differences between specific treatment groups at (**b**) day (**d**) 1, (**c**) d 2; (**d**) d 3, (**e**) d 4, (**f**) d 6 and (**g**) d 8 post-hatch. F – chicks feed immediately post-hatch; NF – chicks without access to feed for the first 48 h post-hatch

**Fig. 7:**
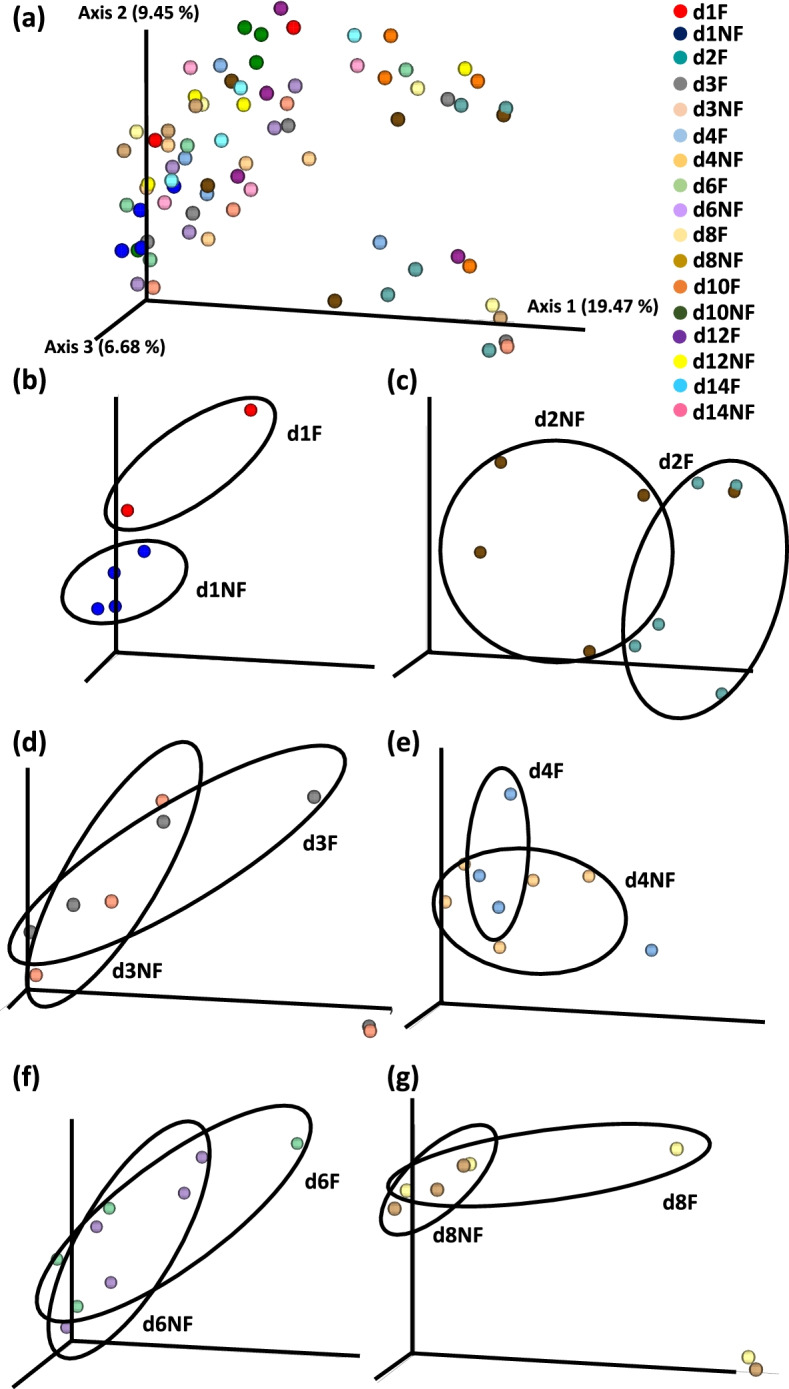
Effect of delay in feed access for the first 48 h post-hatch on beta diversity of cecal mucosal bacterial population from day 1 (24 h) through day 14 (336 h) post-hatch using the Principal Coordinate analysis (PCoA) based on the Unweighted UniFrac distances between groups. Panel (**a**) shown PCoA for all samples while (**b**-**g**) panels depict differences between specific treatment groups at (**b**) day (**d**) 1, (**c**) d 2; (**d**) d 3, (**e**) d 4, (**f**) d 6 and (**g**) d 8 post-hatch. F – chicks feed immediately post-hatch; NF – chicks without access to feed for the first 48 h post-hatch

PERMANOVA analysis based on the Unweighted UniFrac distance matrix was used to determine the similarities between pairs of microbial communities (Table 3). Three bacterial communities, IL-L, CE-L, and CE-M exhibited a Time × TRT interaction (Table 3). Principal coordinate analysis (PCoA) based on the Unweighted UniFrac distance matrix indicated differences in bacterial populations in IL-L, CE-L, and CE-M (Figs. 5, 6 and 7). No differences in beta diversity were detected for IL-M bacterial population as shown by PERMANOVA analysis (Table 3) and PCoA (data not shown). Differences in bacterial communities in IL-L are shown in Fig. 5 with panel (a, Fig. 5a) showing PCoA for all samples, while panels (b-g) show separate comparisons between (b) d1 F and d2 F, or (c-g) F and NF populations at remaining time points. Due to lack of digesta in the ileum of NF birds for the first 48 h, it was impossible to determine bacterial communities. Separation of bacterial communities of F birds between day 1 and 2 PH was observed in IL-L (Fig. 5b). Lack of feed for the first 48 h PH led to clear clustering of bacterial communities between F and NF birds up to day 3 PH (Fig. 5c-g). In the CE-L bacterial population, lack of feed resulted in separation of bacterial communities between F and NF birds up to 8 days PH (Fig. 6a-g). In the CE-M bacterial communities (Fig. 7a-g), distinguished clustering of bacteria due to lack of feed was detected between F and NF birds on day 1 and 2 PH (Fig. 7b and c).

**Table 3:** PERMANOVA *p*-values for interactive [Time x TRT (fed or delayed access to feed)] or main (Time and TRT) effects for bacterial community beta diversity (PERMANOVA results) in ileal (IL) and cecal (CE) luminal (L) and mucosal (M) samples collected from days 1 (24 h) to 14 (336 h) post-hatch

	Pr = F			
	IL-L	IL-M	CE-L	CE-M
Unweighted UniFrac Distance				
Time	0.001	0.082	0.001	0.002
TRT	0.255	0.110	0.307	0.004
Time × TRT	0.001	0.113	0.001	0.001

### Taxonomic composition

**Fig. 8:**
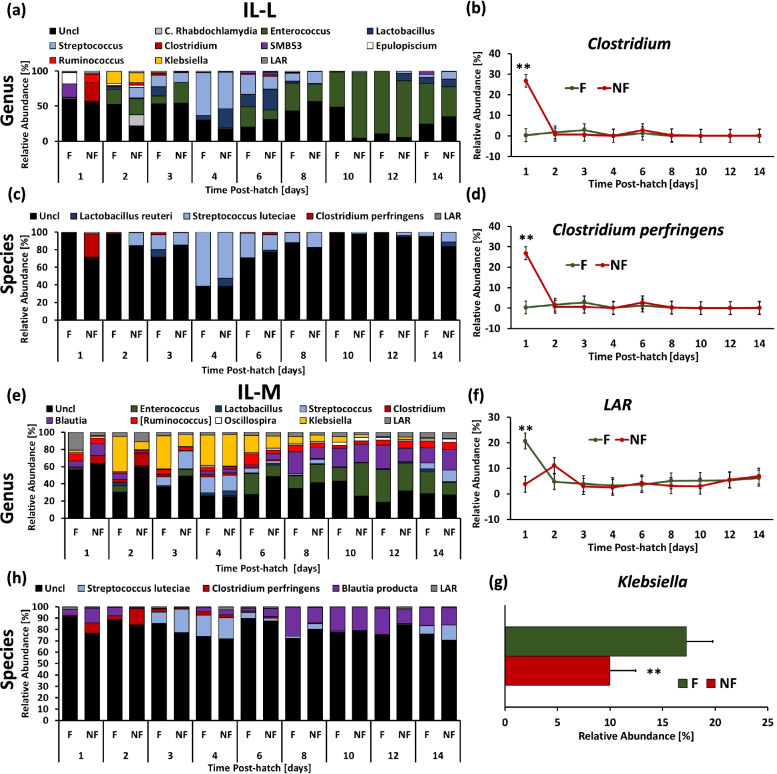
Effect of delay in feed access for the first 48 h post-hatch on relative bacterial abundance (%) in ileal luminal (IL-L, a-d) and mucosal (IL-M, e–f) bacterial populations from day 1 (24 h) through day 14 (336 h) post-hatch at genus and species level. Taxonomic profile of chicken IL-L at (**a**) genus and (**c**) species level. Effect of delayed access to feed early post-hatch on (**b**) *Clostridium* and (**d**) *Clostridium perfringens* level in IL-L. Taxonomic profile of chicken IL-M at (**e**) genus and (**h**) species level. Effect of delayed access to feed early post-hatch on (**f**) Low Abundant Reads (LAR) and (**g**) *Klebsiella* level in IL-M. Asterisk denote statistically significant (*P* < 0.05) differences between fed (F) and not-fed (NF) chickens

**Fig. 9:**
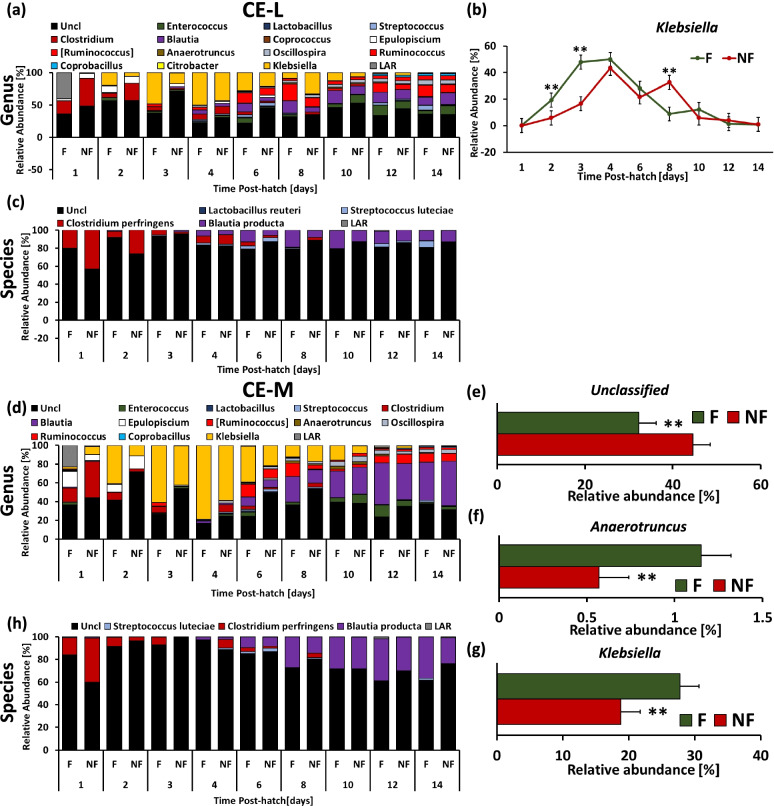
Effect of delay in feed access for the first 48 h post-hatch on relative bacterial abundance (%) in cecal luminal (CE-L, a-c) and mucosal (CE-M, d-g) bacterial populations day 1 (24 h) through day 14 (336 h) post-hatch at genus and species level. Taxonomic profile of chicken CE-L at (**a**) genus and (**c**) species level. Effect of delayed access to feed early post-hatch on (**b**) *Klebsiella* level in CE-L. Taxonomic profile of chicken CE-M at (**d**) genus and (**h**) species level. Effect of delayed access to feed early post-hatch on (**e**) Unclassified bacteria (Uncl), (**f**) *Anaerotruncus* and (**g**) *Klebsiella* level in CE-M. Asterisks denote statistically significant differences between fed (F) and not-fed (NF) chickens (*P* < 0.05)

**Fig. 10:**
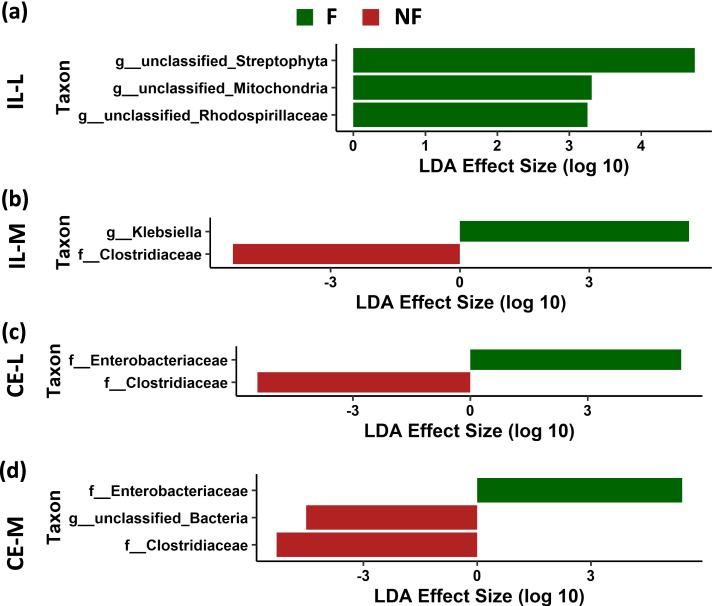
Effect of delay in feed access for the first 48 h post-hatch on differentially abundant bacterial taxa as determined by Linear Discriminant Analysis (LDA) effect size (LEfSe) analysis in ileal luminal (IL-L, **a**) and mucosal (IL-M, **b**), and cecal luminal (CE-L, **c**) and mucosal (CE-M, **d**) bacterial population 2 or 3 days post-hatch. F-chicken immediately fed after hatch, NF – chicken with 48 h delayed access to feed

Taxonomic composition of bacterial communities in IL-L and ILM, and CE-L and CE-C between day 1 and day 14 at the genus and species level is presented in Figs. 8 and 9, respectively. Taxa that were significantly affected by Time × TRT interaction or only affected by TRT (*P* > 0.05 for interaction) are presented in both figures. Significant (*P* < 0.05) developmental effects on genera and species taxa in all four microbial populations are presented in Supplementary Figures (1-4) (developmental effects are only presented for genera and species that exhibited no significant interaction between Time and TRT). The differentially abundant taxa were found using LEfSe analysis (Fig. 10). Figure 8 presents the taxonomic profile of IL-L (Fig. 8a and c) and IL-M (Fig. 8e and h) at the genus and species level, respectively. In IL-L, only *Clostridium* on genus level (Fig. 8b) and *C. perfringens* on species level (Fig. 8d) had a Time × TRT interaction (*P* < 0.05), where 1 day PH NF birds had significantly (*P* < 0.05) higher level of *Clostridium* and *C. perfringens* in comparison to F birds. In IL-M, the level of low abundance reads (LAR) that represents bacterial taxa characterized by very low abundance were significantly higher (*P* < 0.05) in F birds in comparison to NF birds at day 1 PH (Fig. 8f), while at species level (Fig. 8g) the abundance of *Klebsiella* was higher (*P* < 0.05) in F birds than in NF birds. Developmental changes in bacterial taxa in IL-L are presented in Supplementary Fig. 1. Decrease in abundance during development was observed for Unclassified bacteria and *Ruminococcus* (Fig. S2a and e, respectively), while *Entercococcus* abundance increased at the end of the second week PH (Fig. S2b). For *Lactobacillus* and *Streptococcus*, relative abundance saw an increase at the end of the first week PH followed by a steep decrease after reaching a peak (Fig. S2 c and d), while transient increase in *Klebsiella* were observed on 2 day PH (Fig. S2f). At the species level, stable abundance of Unclassified bacteria was observed throughout the first two weeks PH except at 96 h PH. During this time, a significant (*P* < 0.05) drop in Unclassified bacteria abundance was detected (Fig. S2g). The abundance profile of *Streptococcus luteciae* was similar to that of *Streptococcus* (Fig. S2h). In IL-M, abundance of Unclassified bacteria decreased (*P* < 0.05) over time (Fig. S3a), while the abundance of *Entercococcus* and *Oscillospira* increased during the second week PH (Fig. S3b and e). Abundance of *Streptococcus* and *Klebsiella* were higher (*P* < 0.05) during the first week PH (Fig. S3c and f). A lower level of *Blautia* relative abundance was observed between 48 and 144 h followed by an increase between 192–336 h PH (Fig. S3d). The abundance profiles of *S. luteciae* and *Blautia producta* were similar to their abundance at the genus level (Fig. S3g and h).

Taxonomic profiles of bacterial communities in CE-L and CE-M are presented in Fig. 9 at genus (Fig. 9a and d) and species (Fig. 9c and h) level. In CE-L, only *Klebsiella* abundance was affected by time and TRT (*P* < 0.05) with its relative abundance significantly higher at 2 and 3 days PH (*P* < 0.05) in F birds in comparison to NF birds (Fig. 9b). In contrast, at 192 h PH NF birds were characterized by higher (*P* < 0.01) abundance of *Klebsiella* than F birds. None of the species identified in CE-L were affected by time and TRT interaction or TRT (Fig. 9c). In CE-M (Fig. 9d and h), the relative abundance of Unclassified bacteria (Fig. 9e), *Anaerotruncus* (Fig. 9f) and *Klebsiella* (Fig. 9g) were significantly (*P* < 0.01) affected by access to feed early PH (TRT) with the level of Unclassified bacteria higher in NF birds, and the level of *Anaeroutruncus* and *Klebsiella* elevated in F birds in comparison to NF birds. Similar to CE-L, no identified species in CE-M were affected by Time and TRT interaction or TRT alone (Fig. 9h). Most of the genera and species in CE-L and CE-M were affected by time (development) regardless of the PH treatment (Supplementary Fig. S4 and Fig. S5, respectively). In CE-L, a decrease in Unclassified bacteria was observed between 4–8 days PH in comparison to day 2 and 3 (Fig. S4a). The relative abundance of *Enterococcus*, *Blautia*, *Anaerotruncus*, *Oscillospira*, *Ruminococcus* and *Coprobacillus* were characterized by an increase during the second week PH (Fig. S4b, e, and h-k, respectively) while *Clostridium*, *Epulopiscium* and LAR were characterized by higher abundance (*P* < 0.05) during first 1–2 days PH followed by a decrease (*P* < 0.05) (Fig. S4d, f, and l, respectively). The relative abundance of *Streptococcus* and [*Ruminococcus*] showed sinusoidal changes during the second week PH (Fig. S4c and g, respectively). Square brackets indicate possible misclassification by taxonomy database. At the species level of CE-L, the relative abundance of Unclassified bacteria showed stable levels during the first two weeks PH except at day 3, when a slight increase (*P* < 0.05) of Unclassified bacteria was observed in comparison to day 1 (Fig. S4m). The relative abundance profile of *S. lutecia* (Fig. S4n), *C. perfringens* (Fig. S4o) and *B. producta* (Fig. S4p) followed the taxonomic profile of the respective genera. In CE-M, the relative abundance level of *Enterococcus* and *Anaerotruncus* showed increase during the second week PH followed by the decrease (Fig. S5a and f, respectively). *Clostridium* and LAR abundance profiles were characterized by early PH decrease (*P* < 0.05) followed by steady low levels (Fig. S5c and k, respectively). Relative abundance of *Blautia* (Fig. S5d), *Oscillospira* (Fig. S5g), *Ruminococcus* (Fig. S5h) and *Coprobacillus* (Fig. S5i) increased (*P* < 0.05) during second week PH. An increase in relative abundance of *Streptococcus* 6 days and *Klebsiella* 2–6 days PH was observed (Fig. S5b and j, respectively). The abundance level of [*Ruminococcus*] was elevated at 6–8 and 12–14 days PH in comparison to 1–2 and 4 days PH (Fig. S5e). At species level, an increase in Unclassified bacteria between 2–4 days PH was detected, while the abundance profiles of *S. luteciae*, *C. perfringenes* and *B. producta* were similar to their respective genera (Fig. S5l-o).

To evaluate the significance of differential bacterial abundance between F and NF birds early PH (2–3 days PH), LEfSe analysis was performed in all four bacterial communities (Fig. 10). In IL-L, unclassified *Streptophyta*, mitochondria and Rhodospirillaceae had greater relative abundance in F birds, while in IL-M, *Klebsiella* was more abundant in F birds and Clostridiaceae was more abundant in NF birds (Fig. 10a and b). In CE-L, family Enterobacteriaceae and Clostridiaceae were more abundant in F and NF birds, respectively (Fig. 10c). In CE-M, family Enterobacteriaceae was more abundant in F birds, while Unclassified bacteria and family Clostridiaceae were more abundant in NF birds (Fig. 10d).

### Predicted function of the microbiota

The overall comparison of predicted functions of microbiota in F and NF birds in IL-L, IL-M, CE-L, and CE-M are presented in Supplementary Figure S6. The least amount of predicted metabolic pathways that were significantly (*P* < 0.05) different between F and NF birds were present in IL-M (Fig. S6b) while the CE-M population was characterized by the highest number of predicted metabolic pathways that differed between F and NF birds (Fig S6d). In IL-L, among identified metabolic pathways were the urea cycle, nitrate reduction and L-histidine degradation I which were significantly different in birds with immediate access to feed PH (Fig. S6a). In IL-M, octane oxidation was the only pathway significantly (*P* < 0.05) different between F and NF birds (Fig. S6b). In CE-L, almost twice the number of pathways was significantly different in NF birds in comparison to F birds, including amino acids biosynthesis pathways, glycogen degradation pathway and pyruvate/isobutanol fermentation pathway (Fig. S6c). In contrast, in CE-M most of the identified metabolic pathways were different between F and NF birds except for toluene degradation IV pathway. The top differential pathways between F and NF birds were related to L-histidine and chitin derivatives degradation and thiazole and phylloquinol biosynthesis (Fig. S6d).

## Discussion

Although, the microbiota of the chicken intestine has been evaluated during the first days and weeks PH, the effects of current poultry production systems involving delayed access to feed early PH on the microbiota development and maturation have not been investigated. Furthermore, most research on the chicken microbiota has focused only on the luminal populations without exploring the microbiota attached to epithelial cells (mucosal populations). In this manuscript, we focused on the effect of delayed feeding early PH on microbiota development and maturation, and determined the developmental changes in luminal and mucosal microbiota in both ileum and ceca. Microbes attached to GIT epithelium have their own biological role [25] and should be analyzed separately from those in the intestinal lumen. Moreover, we revisited the idea that chicken embryos are not sterile and that their GIT is already colonized during embryonic development.

Analysis of early microbiota samples collected from embryos 48 h before hatch, at hatch, and chicks 4 h PH revealed the presence of low level of bacterial reads in ileum and cecum. Moreover, microbiota in ileum samples seemed to be more developed at these early time points with higher ASVs, richness, evenness, and Shannon index than the in the ceca. Most of the bacteria detected in early microbiota could not be identified in any more detail than the kingdom level (60% and 90% in ileum and cecum, respectively). Our results support the data of others [11, 13] indicating that the chicken embryo is not sterile and its GIT has been already colonized. The source of the microbiota in late embryos and hatched chicks is probably derived from the maternal bacteria deposited in the egg [11, 13]. However, it should be mentioned that Richards-Rios et al. [10] were not able to detect any bacterial reads in the embryonic GIT at day 18 using PCR amplicons and gel electrophoresis. We found that most of the bacteria detected in the ileum belonged to phylum Firmicutes, family Clostridiaceae and Enterobacteriaceae, while we were only able identify the reads as bacterial reads in cecal samples from late embryos and newly hatched chicks. It has been shown that Enterobacteriaceae can be acquired before hatch from the oviduct of the hen or from the environment through pores in the eggshell [12, 50]. The presence of Clostridiaceae, a family of obligate anaerobes, is more questionable, however it has been shown that the source of Clostridiaceae is usually environmental [51] and that not all Clostridiaceae are true obligate anaerobes [52]. Moreover, presence of Clostridiaceae in one day old turkeys has been shown previously [53]. Presence of *Enterococcus* in late embryo and newly hatched chicks suggest their positive effects on the microbiota since this genus was shown to exhibit polysaccharide-degrading activity, and promote *Lactobacillus* colonization, and is associated with positive morphological changes in the GIT [54, 55]. Additionally, Akinyemi et al. [56] have shown changes in embryo microbiota across developmental stages, with reduction of the microbial population at day 19 of embryonic development and some microbes disappearing before hatch.

After hatch, chicks are exposed to a diverse range of bacteria first encountered in hatcheries, and from animal handlers, and later from the housing environment, litter, feed and water. In our study, half of newly hatched chicks were immediately exposed to feed after hatch, while the second half were maintained in conditions similar to standard poultry rearing and delayed access to feed for 48 h. Because newly hatched chicks are exposed first to environmental, non-avian sources of bacteria, the colonization pattern of GIT has been shown to be highly variable [17] and characterized by low diversity, high instability and susceptibility to modification by exogenous factors [57, 58]. Our data indicates that in most cases the alpha diversity indices were lower in NF birds in comparison to F birds early post-hatch (2–3 days PH). Later during development, any microbiota changes that were observed were opposite to those found in early PH time points, with NF birds characterized by higher alpha-diversity. High alpha diversity of gut microbiota has been shown to be favorable to overall animal health and productivity in pigs [59]. Similar to a previous report [60], a decrease in ileum alpha diversity and an increase in cecal alpha diversity were observed in the present study after hatch. It has been shown previously that higher diversity in the ceca is due to low passage rate of the digesta, lower pH, and the presence of small and soluble particles [3, 28]. Moreover, the mucosal populations have been characterized by higher diversities in comparison to luminal ones, as shown before [25]. The increase in alpha diversity in NF in comparison to F birds later during development could be a way of compensation due to interrupted microbiota development early PH. Among all four bacterial populations, only IL-M was not affected at all by the delayed access to feed early PH. It has been shown that the first bacteria entering the intestine are able to adhere to the epithelial cells without competition, rapidly establish its presence and therefore have the highest influence on the subsequent development of intestinal microbiota [2, 17]. We can speculate that the environmental bacteria, (acquired from the incubator, water, or cages), that first entered the intestine were not influenced by access to feed, since both bacterial populations from F and NF birds in IL-M, have similar alpha-diversity in contrast to IL-L or cecal bacterial populations. Similar to alpha diversity, beta diversity was only affected in IL-L, CE-L, and CE-M, while IL-M was not affected by delayed access to feed. In all bacterial populations, which were affected by delayed access to feed, clear separation between microbiota of F and NF birds was detected; however, the length of the differential clustering was different, lasting only for a day or two in IL-L and in CE-L, and up to 8 days PH in CE-M. Our results clearly show that delayed access to feed early PH affects the structure of the microbiota, as shown by differences in beta diversity. These differences in bacterial diversity are probably due to limited exposure of NF chickens to microbes coming from the feed, and this can have long-lasting consequences. Restriction to microbial exposure in early life has been shown to have an impact on mucin production [61]. Our earlier study showed downregulation of the MUC2 gene in the ileum [43] but not in the ceca [44]. Moreover, disturbances in intestinal microbiota have been shown to delay growth, weaken the host resistance, and increase the host susceptibility to various infections [62]. Colonization patterns are unstable in young birds, including newly hatched chicks, making them more susceptible to bacterial infection [61], leading to negative effects on intestinal maturation and integrity. Additional disturbances due to lack of feed early PH may contribute to unstable colonization patterns, affecting intestinal immune responses. Establishment of adequate microbiota is an effective barrier against opportunistic pathogens, can provide metabolites to the animal, and properly stimulate the immune system [63]. Different responses of the four bacterial populations (IL-L, IL-M, CE-L and CE-M) strongly support the advantage of focusing the research effort on these populations separately. Differences in the response to delayed access to feed early PH could be related to the respective functions of each region of the GIT and the role of mucosal bacteria. The microbiota in the small intestine have been shown to contribute to its function in digestion and nutrient absorption, while the cecal microbiota have been shown being responsible for protecting birds against bacterial infections [64].

The establishment of microbiota in the GIT in young animals is characterized very often by high turnover of many transient species and large changes in community structure [65] due to resource competition between bacterial species, shift in host diets and age-related variation in the GIT [66, 67]. The GIT is first colonized by facultative anaerobes that reduce the oxygen level in the GIT allowing for the establishment of subsequent anaerobic bacteria [66]. The shift from facultative anaerobes to strict anaerobes in the chicken GIT takes place around 7 days PH [68]. The first species colonizing the GIT have been shown to have a pronounced effect on the establishment of intestinal microbiota [2, 48, 68]. We have observed similar pattern of colonization, with *Klebsiella* (aerobic or facultative anaerobic), *Streptococcus* (facultative anaerobic), *Enterococcus* (facultative anaerobe) and *Lactobacillus* (oxygen tolerant) been predominant during first week PH, followed by obligate anaerobes such as *Ruminococcus*, *Blautia*, *Oscillospira*, *Coprobacillus*, *Anaerotruncus* and *Epulopiscium* during second week PH. Moreover, it has been shown that the bacterial succession in the GIT at a very early stage of life can be influenced by bacterial composition of inocula [69], inoculation with microbiota from adults [70], or contact with the hen [18]. Similar to previous reports [22, 24, 68], we observed transient changes in taxonomic composition in all four bacterial populations at every level. Consistent with other studies [71, 72], ileal and cecal microbiota were predominantly composed by Firmicutes and Proteobacteria. However, Ballou et al. [68] determined that chicken microbiota early PH are dominated by Gammaproteobacteria, while Kers et al. [51] showed similar to our results, that Clostridiaceae are predominant in young chickens. The taxonomic composition during development in the ileum and ceca could be influenced by many factors including feed and environmental factors [18, 60]. Even though only a small portion of the mapped reads could be identified at the species level, we have also shown transient changes in bacterial species in ileal and cecal bacterial populations. Our results clearly support previous reports that microbiota residing in the GIT of chickens remains largely unexplored with more than 200 species isolated from the GIT [2, 25, 73].

The taxonomic changes due to lack of feed for the first 48 h PH were smaller than expected. However, in IL-L, relative abundance of *C. perfringens* was elevated in NF birds. As discussed above, presence of Clostridiaceae family in early PH chicks has been observed previously [51]. *C. perfringens* has been associated with dysbacteriosis and bacterial enteritis under certain conditions. While it is a commensal microorganism under normal conditions, it can become an opportunistic pathogen under certain conditions that promote its growth. Here, that the presence of *C. perfringens* in one day old chicks does not indicate bacterial infection, but suggests that in certain circumstances, such as presence of *Eimeria* species in the environment or in the GIT or other pre-disposing factors, NF chicks could be more susceptible to necrotic enteritis infection. In IL-M, birds that had early access to feed (F) were characterized by a higher relative amount of LAR (low abundance reads) indicating that the presence of feed promotes colonization with many bacterial species. Additionally, the level of *Klebsiella* was elevated in F birds. *Klebsiella,* an aerobic bacteria with facultative anaerobic properties belongs to the family Enterobacteriaceae that has been shown to be a part of normal microbiota in the intestine [74]. Similar to IL-M, *Klebsiella* was elevated in F birds in comparison to NF birds also in CE-L and CE-M. The specific role of *Klebsiella* in chicken microbiota is unknown. However, Potturi et al. [75] observed an increase in the presence of aerobic bacteria in ileum of turkey poults with delayed access to feed. Additionally, delayed access to feed early PH increased the relative abundance level of unclassified bacteria in CE-L. In CE-M, the level of *Anaerotruncus* (family Clostridiaceae), an anaerobic and spore forming bacteria [76], was elevated in F birds in comparison to NF birds. *Anaerotruncus* has been shown to express enzymes required for butyrate production [76]. Changes in taxonomic composition were confirmed by LEfSe analysis on day 3 PH between F and NF birds. In general, birds with delayed access to feed were characterized by an increased relative abundance level of Clostridiaceae while F birds were characterized by a higher level of Enterobacteriaceae. Relatively small changes in taxonomic composition in all four bacterial populations due to delayed access to feed early PH, confirm earlier observations of Ballou et al. [68] that age is more influential in microbiota development than other factors.

Similar to diversity and taxonomic composition data, IL-M bacterial population was the least affected regarding predicted function of the microbiota. Most changes in metabolic pathways were observed in IL-L, CE-L and CE-M. Enrichment of urea cycle and nitrate reduction pathways in IL-L of F birds is probably related to normal metabolic changes associated with feed digestion and processing. Amino acid degradation releases nitrogen as ammonia that is relatively toxic to most animals, including birds. [77]. In birds, ammonia is detoxified through synthesis of uric acid for excretion [77]. Bacteria capable of degrading uric acid have been demonstrated in GIT of birds with the end product of this degradation being short chain fatty acids, ammonia, and carbon dioxide [77]. In contrast to IL-L, twice as many pathways were different between NF bird and F birds in CE-L. It is possible that the cecal luminal bacterial populations overcompensate for the lack of feed for the first 48 h PH later in life; however, this needs to be investigated thoroughly. In CE-M, the enrichment of metabolic pathways followed the access to feed after hatch, with most of the metabolic pathways significantly different between F and NF birds. Lack of changes in IL-M could be an example of bacterial resistance to unfavorable environment. Moya and Ferrer [78] suggested taking into consideration bacterial stability, resistance, resilience, and functional redundancy when describing bacterial disturbance.

## Conclusions

The first week PH is critical for microbiota development [48], leaving only a small window for remodeling resulting in permanent microbiota [79, 80]. In our study, we have shown that delayed access to feed early PH affected the microbiota development, especially during the early days PH. The negative effects of delayed feed early PH have been known for years, but only recent data indicates that delayed access to feed PH also has effects on microbiota. Proper development of microbiota could be very important for disease prevention especially in the era of withdrawal of antibiotic growth promoters and increased antibiotic resistance. However, the data suggests that the developmental factor (age) is a much stronger driver of microbiota development than any treatment PH. Moreover, we have shown that mucosal and luminal bacterial populations of the ileum and ceca are unique and respond to treatment differently, strongly suggesting that these two bacterial populations, mucosal and luminal, should be analyzed separately.

## Methods

### Animals and experimental protocols

All animal care procedures were approved by the USDA-ARS Institutional Animal Care and Use Committee. All methods were carried out in accordance with relevant guidelines and regulations. This study was performed and reported in accordance with ARRIVE guidelines (https://arriveguidelines.org/). The full description of the experiment has been published previously [42, 43]. Briefly, two hundred and fifty fertile Ross 708 broiler chicken eggs were obtained from a local hatchery (Perdue Hatchery, Hurlock, MD) and incubated under standard conditions (37.5 °C and 60% humidity) in USDA-ARS facility as described previously [42, 43]. All birds used in this experiment were hatched during a window of 486 and 496 h of incubation. During that time, the hatcher was monitored every 2 h. Birds were removed from the hatcher in three batches (within 180–240 min after occlusion) and randomly distributed between experimental groups in a way that each battery pen included birds from each batch (14–15 hatchlings per battery pen total). Birds were placed into heated battery-brooders equipped with 2 nipple drinkers and one feeder. Feed (standard commercial corn-soybean meal diet) was provided for birds that were fed after hatch when the first batch of birds were placed into battery pens. Hatchlings were divided into two treatment groups randomly (*n* = 6 battery pens for each treatment). One group received feed and water immediately after placement (F) while the second one received water immediately but had delayed access to feed for 48 h (NF) to mimic commercial hatchery operations. Birds were fed a commercial type corn-soybean meal-based starter diet [42, 43].

### Tissue sampling

Birds were sampled at hatch (0 h, wet chicks, within 30 min from hatch), and 4 h (birds selected from the first batch of chicks placed into battery pens), 1 (24 h), 2 (48 h), 3 (72 h), 4 (96 h), 6 (144 h), 8 (192 h), 10 (240 h), 12 (288 h) and 14 (336 h) days after the start of feeding. Additionally, embryos were sampled at embryonic (**e**) day 19 (-48 h, *n* = 6). The sampling times were selected based on previously published data [41] and adjusted to determine changes in microbiota development during first two weeks PH. Since microbiota composition among parts of small intestine (duodenum, jejunum and ileum) has been reported to be similar to each other, we decided to focus only on IL and the CE, as the main microbiome organ in birds [22]. Due to the embryo size, ileal (IL) and cecal (CE) samples were collected from two embryos and pooled together (by tissue type) [42, 43]. Starting at 24 h PH, one chick per pen, selected at random, was sacrificed by cervical dislocation. To determine the luminal (L) and mucosal (M) bacterial populations, at each sampling point the distal part of the ileum (from Meckel’s diverticulum to ileocecal junction) and ceca were dissected for collection of ileal and cecal content (IL-L and CE-L) as well as their epithelial scrapings (IL-M and CE-M), respectively [81]. Isolated specimens were snap-frozen in liquid nitrogen and stored at -80℃ until bacterial DNA isolation [81, 82].

### DNA isolation and library preparation

DNA was extracted from each of the ileal and cecal scrapings and contents and were evaluated as described previously [82]. The 16S rRNA gene amplicon libraries were generated using the workflow and chemistry supplied by Illumina (Illumina, Inc., San Diego, CA) and PCR primers (Forward: 5’-TCGTCGGCAGCGTCAGATGTGTATAAGAGACAGCCTACGGGNGGCWGC AG-3’ and Reverse: 5’-GTCTCGTGGGCTCGGAGATGTGTATAAGAGACAGGACTACHV GGGTATCTAATCC-3’) targeted the V3-V4 variable region of the 16S gene. Amplicon PCR followed by index PCR and PCR amplicon cleaning were performed as described previously [82]. Concentration and quality of the amplicons were determined using Qubit 3 (Thermo Fisher Scientific, Inc) and Bioanalyzer (Agilent Technologies), respectively. The pooled (96 barcoded amplicons) DNA library (4 nM) and PhiX control v3 (Illumina, Inc., 4 nM) were denatured with 0.2 N NaOH (Sigma-Aldrich, Corp., St. Louis, MO) and diluted to a final concentration of 4 pM. The library was mixed with PhiX control (20% v/v) and pair-end 2 × 300-bp sequencing was performed using the Illumina MiSeq platform and a MiSeq Reagent Kit v3 (Illumina, Inc). The 16S rRNA gene sequences determined in this study were deposited in the NCBI Sequence Read Archive (SRA) database (SRA accession # PRJNA779402).

### 16S rRNA gene sequence, data processing and analysis

Quantitative Insight Into Microbial Ecology (QIIME) software package 2 (version 2017.12.0, http://qiime2.org) was used to perform quality control and analysis of the sequence reads [81]. Raw fastq files were demultiplexed using q2-demux and quality filtered and dereplicated with q2-dada2 [81]. Sequences with an average Phred score lower than 25 were removed. Representative sequence sets for each dada2 sequence variant were used for taxonomy classification. MAFFT was used for multiple sequence alignment and Fastree was used to generate phylogenetic trees. Naïve Bayesian classifier was used for taxonomic classification against the Greengenes database v. 13_8 (http://greengenes.secongenome.com) [82]. Data were rarefied to the lowest possible counts of sequences per sample (sequencing depth, Table 1) for calculation of alpha and beta diversities. Alpha diversity indices (ASVs, Shannon’s diversity index, Pielou’s Evenness, Faith’s Phylogenetic Diversity) were obtained through QIIME 2 package. Analysis of beta diversity was performed by QIIME2 employing Unweighted UniFrac. Principal coordinate analysis (PCoA) based on all far distance metrics was implemented in the QIIME2 software. QIIME data were transformed using R package Compositions [83] followed by Phylogenetic Investigation of Communities by Reconstruction of Unobserved States (PICRUSt)2 [84] was used to predict metagenome pathways for each primer set using the MetaCyc database (MetaCyc.org) [85]. Statistical Analysis of Metagenomic Profiles (STAMP) [86] was used to create a visualization of metabolic pathway comparison. Linear Discriminant Analysis (LDA) Effect Size (LEfSe) algorithm was used to identify taxa with significant differential abundance between F and NF birds during the first couple of days post-hatch [87]. Due to the fact the presence of feed in the GIT was one of the factors influencing microbiota development, chloroplast and mitochondria was not removed from the microbiota analysis.

### Statistical analyses

Differences between alpha diversity indices were tested using Kruskal–Wallis test (QIIME2). The difference in community structure due to main effects (time and treatment) and their interaction were statistically tested by non-parametric multivariate ANOVA (PERMANOVA) with 999 permutations using QIIME2 software package. Microbiome composition data were obtained by normalization to total number of reads in each sample (relative abundance) and were analyzed using two-way ANOVA using GLM (SAS). Significance was set at *P* < 0.05. Within STAMP software, a two group comparison was performed using Welsh t-test [88], corrected for false discovery rate (FDR, Benjamin-Hochberg analysis [89]). For the LEfSe analysis, alpha value of 0.5 for Kruskal–Wallis test and the threshold for the log_10_LDA score was set at 2.0.

## Supplementary Information

**Additional file 1:** **Figure S1.** Rarefaction curve in (a) ileal (IL) and cecal (CE) samples collected from chickens from -48 to 4 h post-hatch, and (b) ileal mucosal samples (IL-M), (c) ileal luminal samples (IL-L), (d) cecal mucosal samples (Ce-M), and (e) cecal luminal samples (CE-L) collected from chickens from day 1 (24 h) through day 14 (336 h) post-hatch.

**Additional file 2: Figure S2.** Effect of time (development) on relative bacterial abundance (%) of (a) Unclassified bacteria, (b) *Enterococcus*, (c) *Lactobacillus*, (d) *Streptococcus*, (e) *Ruminococcus*, (f) *Klebsiella,* and (g) Low Abundance reads (LAR) at genus level, and (h) Unclassified bacteria and (i) S*treptococcus luteciae* at species level in ileal luminal bacterial population from day 1 (24 h) through day 14 (336 h) post-hatch. Different letters denote statistically significant (*P*<0.05) differences.

**Additional file 3: Figure S3.** Effect of time (development) on relative bacterial abundance (%) of (a) Unclassified bacteria, (b) *Enterococcus*, (c) *Streptococcus*, (d)*Blautia*, (e) *Oscillospira*, and (f) *Klebsiella,* at genus level, and (g) S*treptococcus luteciae* and (h) *Blautia product *at species level in ileal mucosal bacterial population from day 1 (24 h) through day 14 (336 h) post-hatch. Different letters denote statistically significant (*P*<0.05) differences.

**Additional file 4: Figure S4.** Effect of time (development) on relative bacterial abundance (%) of (a) Unclassified bacteria, (b) *Enterococcus*, (c) *Streptococcus*, (d) *Clostridium*, (e) *Blautia*, (f) *Epulopiscium*, (g) [*Ruminococcus*], (h) *Anaerotruncus*, (i) *Oscillospira*, (j) *Ruminococcus*, (k) *Coprobacillus*, and (l) Low Abundance Reads (LAR) at genus level, and (m) Unclassified bacteria, (n) S*treptococcus luteciae, (o) Clostridium perfringens* and (p) *Blautia product *at species level in cecal luminal bacterial population from day 1 (24 h) through day 14 (336 h) post-hatch. Square brackets are used by taxonomic databases to indicate misclassification of genus. Different letters denote statistically significant (*P*<0.05) differences.

**Additional file 5: Figure S5. **Effect of time (development) on relative bacterial abundance (%) of (a) *Enterococcus*, (b) *Streptococcus*, (c) *Clostridium*, (d) *Blautia*, (e) [*Ruminococcus*], (f) *Anaerotruncus*, (g) *Oscillospira*, (h) *Ruminococcus*, (i) *Coprobacillus*, (j) *Klebsiella* and (k) Low Abundance Reads (LAR) at genus level, and (l) Unclassified bacteria, (m) S*treptococcus luteciae, *(n) *Clostridium perfringens* and (o) *Blautia product *at species level in cecal luminal bacterial population from day 1 (24 h) through day 14 (336 h) post-hatch. Square brackets are used by taxonomic databases to indicate misclassification of genus. Different letters denote statistically significant (*P*<0.05) differences.

**Additional file 6:** **Figure S6.** Effect of delay in feed access for the first 48 h post-hatch on predicted function of the (a) ileal luminal (IL-L), (b) ileal mucosal (IL-M), (c) cecal luminal (CE-L) and (d) cecal mucosal (CE-M) bacterial population from day 1 (24 h) through day 14 post-hatch. Function of the microbiota was determined using PICRUST with MetaCyc database and visualized using STAMP. F-chicken immediately fed after hatch, NF – chicken with 48 h delayed access to feed.

## Data Availability

The 16S rRNA gene sequences determined in this study were deposited in the NCBI Sequence Read Archive (SRA) database (http://nih.gov/bioproject/browse; SRA accession # PRJNA779402).

## Abbreviations

ASV: Amplicon Sequence Variant
CE: Cecum
CE-L: Cecal luminal bacterial population
CE-M: Cecal mucosal bacterial population
F: Fed birds
FDR: False Discovery Rate
GIT: Gastrointestinal tract
IL: Ileum
IL-L: Ileal luminal bacterial population
IL-M: Ileal mucosal bacterial population
L: Luminal
LAR: Low abundance reads
LEfSe: Linear Discriminant Analysis (LDA) Effect Size
M: Mucosal
NF: Not fed birds
PCoA: Principal Coordinate Analysis
PERMANOVA: Non-parametric multivariate ANOVA
PH: Post-hatch
PICRUSt: Phylogenetic Investigation of Communities by Reconstruction of Unobserved States
QIIME: Quantitative Insight Into Microbial Ecology
SRA: NCBI Sequence Read Archive
TRT: Treatment
