# Supplementary figures and images for: Delayed access to feed early post-hatch affects the development and maturation of gastrointestinal tract microbiota in broiler chickens
Source: BMC Microbiol. 2022 Aug 24;22:206. doi: 10.1186/s12866-022-02619-6 (PMC9404604; doi:10.1186/s12866-022-02619-6)

## Slide 1
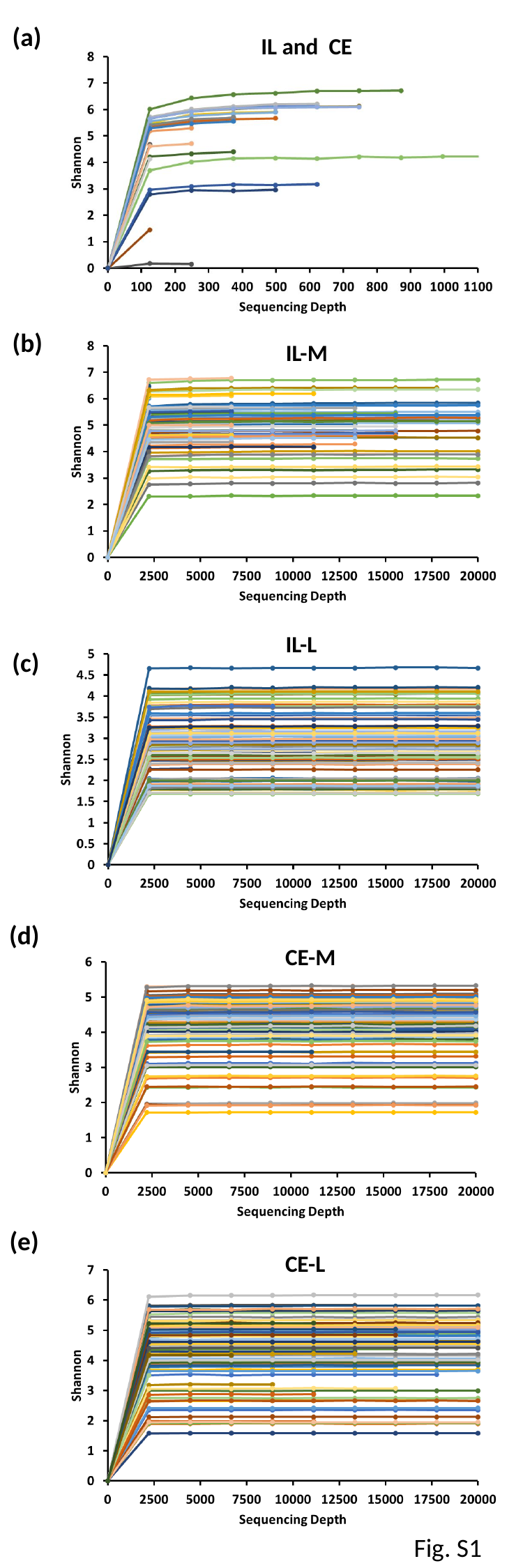

(a)
IL and CE
(b)
IL-M
IL-L
(c)
(d)
CE-M
(e)
CE-L
Fig. S1

Supplement: Supplementary file 1 — Additional file 1: Figure S1. Rarefaction curve in (a) ileal (IL) and cecal (CE) samples collected from chickens from -48 to 4 h post-hatch, and (b) ileal mucosal samples (IL-M), (c) ileal luminal samples (IL-L), (d) cecal mucosal samples (Ce-M), and (e) cecal luminal samples (CE-L) collected from chickens from day 1 (24 h) through day 14 (336 h) post-hatch. [file 12866_2022_2619_MOESM1_ESM.pptx]

## Slide 1
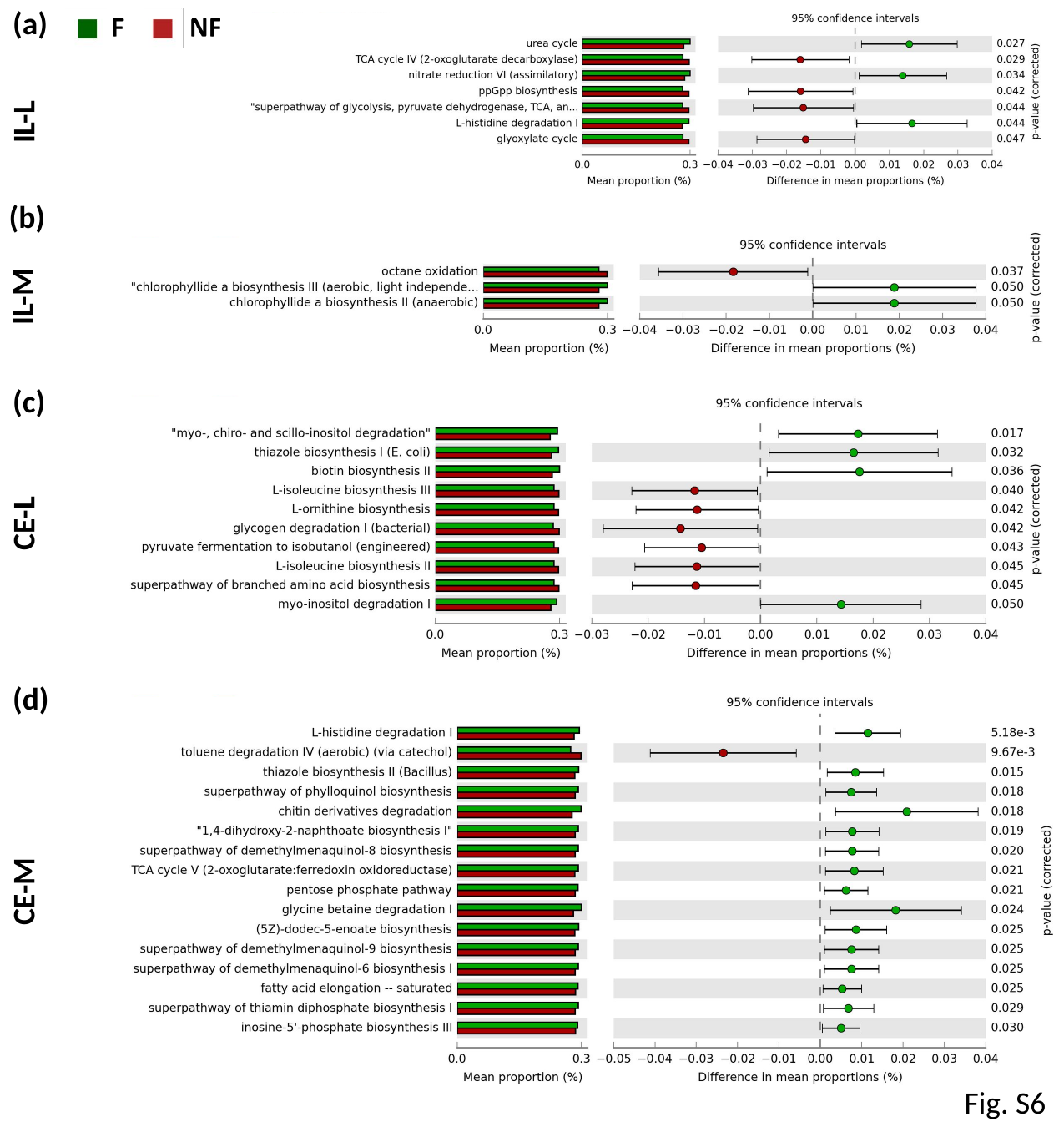

(a)
IL-L
(b)
IL-M
(c)
CE-L
(d)
CE-M
Fig. S6

Supplement: Supplementary file 6 — Additional file 6: Figure S6. Effect of delay in feed access for the first 48 h post-hatch on predicted function of the (a) ileal luminal (IL-L), (b) ileal mucosal (IL-M), (c) cecal luminal (CE-L) and (d) cecal mucosal (CE-M) bacterial population from day 1 (24 h) through day 14 post-hatch. Function of the microbiota was determined using PICRUST with MetaCyc database and visualized using STAMP. F-chicken immediately fed after hatch, NF – chicken with 48 h delayed access to feed. [file 12866_2022_2619_MOESM6_ESM.pptx]
