# Supplementary material for: Delayed access to feed early post-hatch affects the development and maturation of gastrointestinal tract microbiota in broiler chickens
Source: BMC Microbiol. 2022 Aug 24;22:206. doi: 10.1186/s12866-022-02619-6 (PMC9404604; doi:10.1186/s12866-022-02619-6)
Supplement: Supplementary file 2 — Additional file 2: Figure S2. Effect of time (development) on relative bacterial abundance (%) of (a) Unclassified bacteria, (b) Enterococcus, (c) Lactobacillus, (d) Streptococcus, (e) Ruminococcus, (f) Klebsiella, and (g) Low Abundance reads (LAR) at genus level, and (h) Unclassified bacteria and (i) Streptococcus luteciae at species level in ileal luminal bacterial population from day 1 (24 h) through day 14 (336 h) post-hatch. Different letters denote statistically significant (P<0.05) differences. [file 12866_2022_2619_MOESM2_ESM.pptx]

## Slide 1
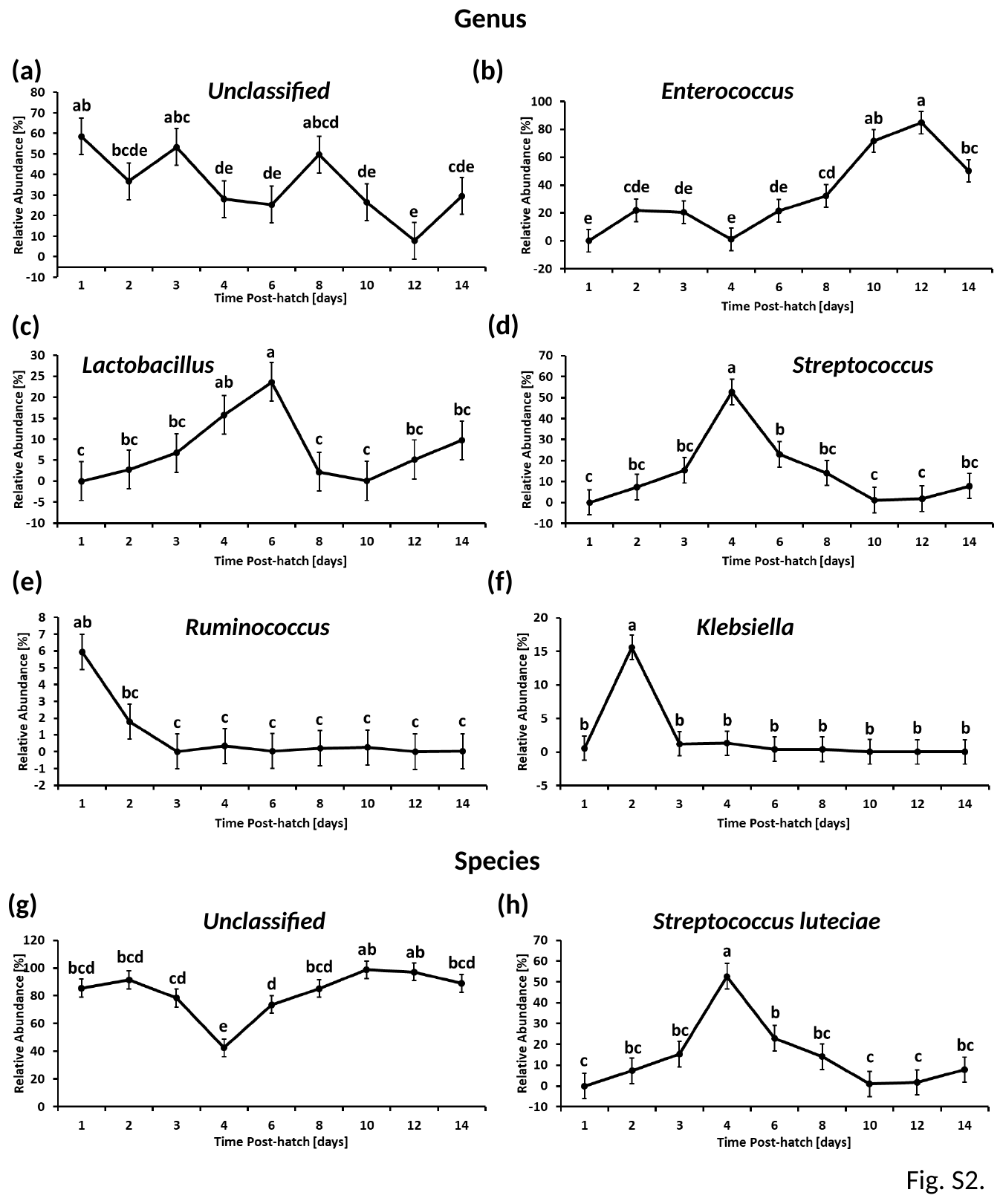

Genus
(a)
(b)
Unclassified
Enterococcus
(d)
(c)
Lactobacillus
Streptococcus
(e)
(f)
Ruminococcus
Klebsiella
Species
(g)
(h)
Unclassified
Streptococcus luteciae
Fig. S2.
