# Supplementary material for: Delayed access to feed early post-hatch affects the development and maturation of gastrointestinal tract microbiota in broiler chickens
Source: BMC Microbiol. 2022 Aug 24;22:206. doi: 10.1186/s12866-022-02619-6 (PMC9404604; doi:10.1186/s12866-022-02619-6)
Supplement: Supplementary file 3 — Additional file 3: Figure S3. Effect of time (development) on relative bacterial abundance (%) of (a) Unclassified bacteria, (b) Enterococcus, (c) Streptococcus, (d)Blautia, (e) Oscillospira, and (f) Klebsiella, at genus level, and (g) Streptococcus luteciae and (h) Blautia product at species level in ileal mucosal bacterial population from day 1 (24 h) through day 14 (336 h) post-hatch. Different letters denote statistically significant (P<0.05) differences. [file 12866_2022_2619_MOESM3_ESM.pptx]

## Slide 1
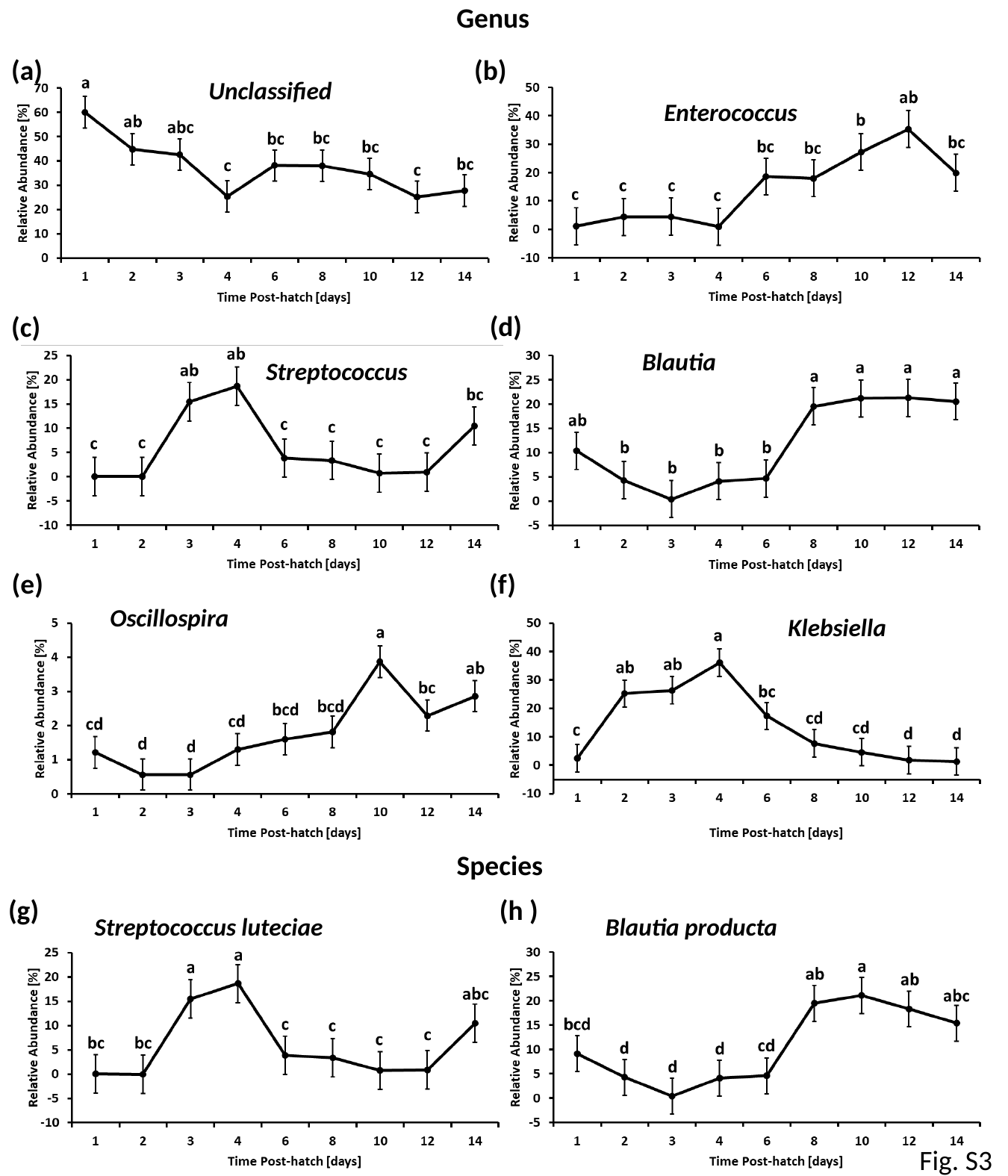

Genus
(a)
(b)
Unclassified
Enterococcus
(d)
(c)
Blautia
Streptococcus
(e)
(f)
Oscillospira
Klebsiella
Species
(g)
(h )
Streptococcus luteciae
Blautia producta
Fig. S3
