# Supplementary material for: Delayed access to feed early post-hatch affects the development and maturation of gastrointestinal tract microbiota in broiler chickens
Source: BMC Microbiol. 2022 Aug 24;22:206. doi: 10.1186/s12866-022-02619-6 (PMC9404604; doi:10.1186/s12866-022-02619-6)
Supplement: Supplementary file 5 — Additional file 5: Figure S5. Effect of time (development) on relative bacterial abundance (%) of (a) Enterococcus, (b) Streptococcus, (c) Clostridium, (d) Blautia, (e) [Ruminococcus], (f) Anaerotruncus, (g) Oscillospira, (h) Ruminococcus, (i) Coprobacillus, (j) Klebsiella and (k) Low Abundance Reads (LAR) at genus level, and (l) Unclassified bacteria, (m) Streptococcus luteciae, (n) Clostridium perfringens and (o) Blautia product at species level in cecal luminal bacterial population from day 1 (24 h) through day 14 (336 h) post-hatch. Square brackets are used by taxonomic databases to indicate misclassification of genus. Different letters denote statistically significant (P<0.05) differences. [file 12866_2022_2619_MOESM5_ESM.pptx]

## Slide 1
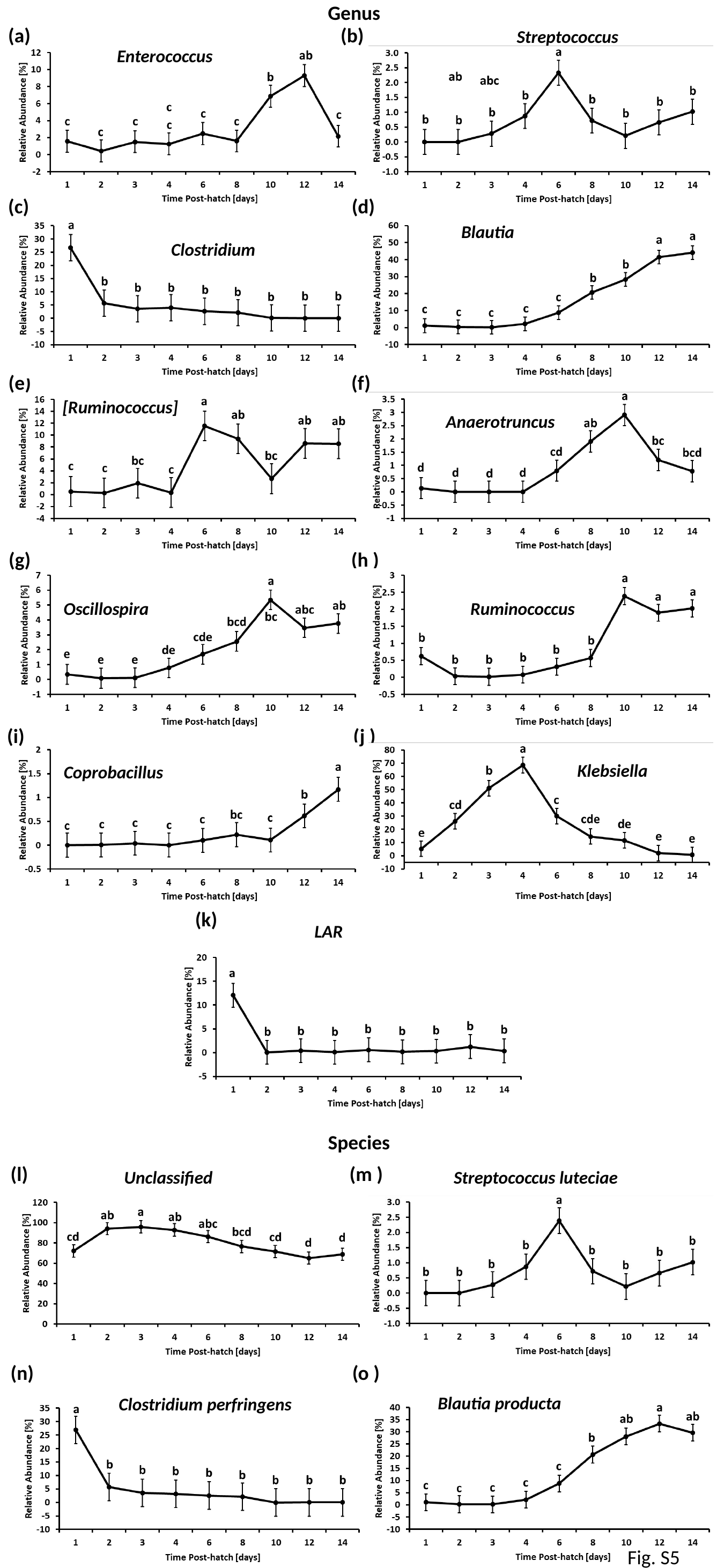

Genus
(a)
(b)
Streptococcus
Enterococcus
(d)
(c)
Blautia
Clostridium
(e)
(f)
[Ruminococcus]
Anaerotruncus
(g)
(h )
Oscillospira
Ruminococcus
(i)
(j )
Klebsiella
Coprobacillus
(k)
LAR
Species
(l)
(m )
Streptococcus luteciae
Unclassified
(n)
(o )
Blautia producta
Clostridium perfringens
Fig. S5
